# Gallstone Disease and Its Association with Hypothyroidism: A Case-Control Study from a Tertiary Care Centre in Puducherry, South India

**DOI:** 10.7759/cureus.112981

**Published:** 2026-07-19

**Authors:** Surya Ram, Jacob Jayakar, Sivaa R

**Affiliations:** 1 General Surgery, Pondicherry Institute of Medical Sciences, Puducherry, IND; 2 Biochemistry, Pondicherry Institute of Medical Sciences, Puducherry, IND

**Keywords:** bile cholesterol supersaturation, biliary stasis, case-control study, cholelithiasis, gallbladder motility, gallstone disease, hypothyroidism, south india, thyroid-stimulating hormone (tsh)

## Abstract

Background and objective: Biliary stasis is a principal driver of gallstone formation. Hypothyroidism promotes lithogenesis through altered lipid metabolism, sphincter of Oddi dysfunction, and impaired gallbladder contractility, thereby promoting bile cholesterol supersaturation. The objectives of this study were (1) to determine whether a statistically significant association exists between gallstone disease and hypothyroidism and (2) to identify whether gallstone composition correlates with thyroid status.

Methods: A prospective case-control study was conducted in the Department of General Surgery at a tertiary care hospital in Puducherry, India, over 25 months (November 2019-November 2021). Patients with ultrasound-confirmed gallstone disease (cases, n = 47) were compared with gallstone-free patients (controls, n = 84). Serum thyroid-stimulating hormone (TSH) was measured by a third-generation chemiluminescence immunometric assay. Gallstone composition was assessed post-operatively by qualitative biochemical analysis (Liebermann-Burchard reaction). Statistical analysis used IBM SPSS Statistics software version 20 (IBM Corp., Armonk, NY, USA); p < 0.05 was considered significant.

Results: Of 47 cases, 19.1% were hypothyroid versus 0% of controls (χ² = 14.41, p = 0.00015). The Haldane-Anscombe-corrected odds ratio (OR) was 14.88, indicating approximately 15-fold greater odds of gallstone disease in hypothyroid individuals. Mixed-type stones predominated (93.6%), followed by cholesterol-type (4.3%) and pigment-type (2.1%). All nine hypothyroid cases had mixed-type stones. Cases clustered in the 30-39-year age group (48.9%).

Conclusion: Hypothyroidism is significantly associated with gallstone disease. The predominance of mixed stones is mechanistically consistent with bile cholesterol supersaturation driven by thyroid hormone deficiency. TSH screening can be considered to be incorporated into the diagnostic workup for cholelithiasis, particularly in high-prevalence populations.

## Introduction

Gallbladder stones are the most prevalent biliary pathology globally, affecting approximately 10% of adults in Western countries and around 4% of the Indian population [1]. A 2024 systematic review and meta-analysis of 115 studies encompassing more than 32 million participants confirmed a pooled gallstone prevalence of 6.1%, with prevalence rising steadily with age. Despite this epidemiological burden, modifiable and treatable metabolic risk factors, including thyroid dysfunction, remain underappreciated in routine surgical workup for cholelithiasis [2].

Hypothyroidism predisposes to gallstone formation through a convergence of well-characterized pathophysiological mechanisms. Thyroid hormone deficiency reduces CYP7A1-mediated bile acid synthesis, increases primary bile acid hydrophobicity, upregulates lithogenic (LITH) gene expression, and impairs sphincter of Oddi relaxation via thyroxine receptors, collectively driving bile cholesterol supersaturation, impaired gallbladder contractility, and prolonged bile residence time [3-9]. Delayed hepatoduodenal transit further impairs clearance of biliary precipitates. 

The epidemiological association between hypothyroidism and cholelithiasis has been investigated for several decades. Prevalence estimates of thyroid dysfunction in gallstone cohorts range from 8% to 40% across diverse populations [10-15].

Despite this evidence, the relationship between gallstone composition, such as cholesterol, pigment, or mixed, and thyroid status has received limited attention. The present study therefore pursued two objectives: (1) to determine whether a statistically significant association exists between gallstone disease and hypothyroidism in a South Indian surgical cohort, and (2) to characterize gallstone composition in relation to thyroid status, addressing a gap highlighted in recent narrative reviews.

## Materials and methods

Study design and setting

This prospective case-control study was conducted in the Department of General Surgery at Pondicherry Institute of Medical Sciences, a tertiary-care teaching hospital in Puducherry, South India, over 25 months (November 2019-November 2021). The study protocol was approved by the Institutional Ethics Committee (approval number RC/19/95), and written informed consent was obtained from all participants prior to enrolment. The study was conducted in adherence with the principles of the Declaration of Helsinki.

Study population

Cases

All patients aged 18-70 years presenting to the General Surgery Department with ultrasound-confirmed gallstone disease were included.

Controls

The control group comprised patients aged 18-70 years presenting to the same department without gallstone disease.

Exclusion Criteria

Patients with acalculous cholecystitis, known thyroid disease under treatment, ongoing thyroid medication, pregnancy, and those who refused to provide informed consent were excluded.

Sample size calculation

Based on an anticipated odds ratio (OR) of 4.5, an 18% exposure prevalence among cases, and a 1:1 case-to-control ratio, a minimum of 84 participants per group was required to achieve 80% statistical power at a two-sided significance level of α = 0.05. Due to COVID-19-related restrictions, enrolment yielded 47 cases and 84 controls (total n = 131). Sampling was non-random and purposive. The implications of this shortfall are addressed in the Limitations section.

Outcome measurements

Serum thyroid-stimulating hormone (TSH) was measured in all participants using a third-generation chemiluminescence immunometric assay (Immulite® TSH; Siemens Healthcare Diagnostics Inc., Los Angeles, CA, USA) on the Immulite® 1000 analyzer (Siemens Healthcare Diagnostics Inc., Los Angeles, CA, USA). The analytical sensitivity of the assay was 0.004 μIU/mL, and the laboratory reference range was 0.4-4.0 μIU/mL. All serum samples were stored at −20°C until analysis. Participants were classified as: hypothyroid (TSH > 4.0 μIU/mL), hyperthyroid (TSH < 0.4 μIU/mL), or euthyroid (TSH 0.4-4.0 μIU/mL).

Gallstone composition was determined postoperatively. All retrieved stones underwent visual examination (colour, texture, cross-sectional appearance) and qualitative biochemical analysis using the Liebermann-Burchard reaction, which detects cholesterol. Stones were classified as cholesterol-type (≥70% cholesterol content), pigment-type (predominantly calcium bilirubinate, <20% cholesterol), or mixed-type (intermediate composition).

Statistical analysis

Data were recorded on a pre-designed proforma, entered in Microsoft Excel 2019 (Microsoft Corp., Redmond, WA, USA), and analysed using IBM SPSS Statistics software, version 20 (IBM Corp., Armonk, NY, USA). Categorical variables were compared using Pearson’s chi-square test. Because no hypothyroid individuals were identified in the control group, the conventional OR was undefined (division by zero). The Haldane-Anscombe correction (adding 0.5 to all four cells of the 2×2 contingency table) was applied to yield a point estimate of OR and enable comparison with published literature. A two-sided p-value < 0.05 was considered statistically significant.

## Results

**Figure 1:**
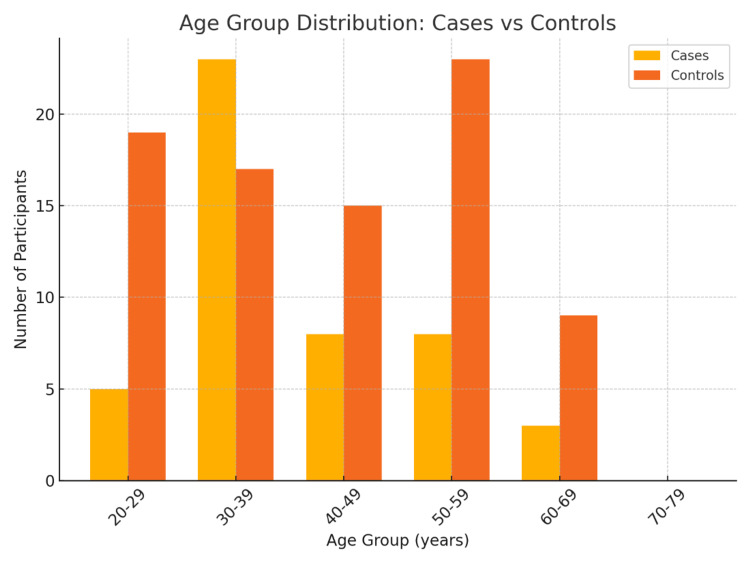
Participants distributed as per their age groups.

A total of 131 participants were enrolled: 47 cases (gallstone disease) and 84 controls (gallstone-free). Gallstone cases clustered in the 30-39 year age group (48.9%, n = 23), whereas controls were more evenly distributed across the 20-59 year range, with a slight peak in the 50-59 year group (Figure 1). This pattern of gallstone disease manifesting predominantly in the third and fourth decades of life aligns with South Indian epidemiological data and may reflect the age of peak hypothyroidism onset in this population [6,16,17].

Thyroid status

Among the 47 cases, nine (19.1%, n = 9) were hypothyroid, and 38 (80.9%, n = 38) were euthyroid; no case was hyperthyroid. All 84 controls were euthyroid (0%, n = 0 hypothyroid). A chi-square test confirmed a strong, statistically significant association between gallstone disease and hypothyroidism (χ² = 14.41, p = 0.00015). Observed and expected frequencies are presented in Table 1.

**Table 1:** Chi-square analysis (χ²): observed and expected frequencies of hypothyroidism χ² = 14.41; df = 1; p = 0.00015

Group	Thyroid Status	Observed (n)	Expected (n)
Cases	Hypothyroid	9 (19.1%)	3.23
Cases	Non-hypothyroid	38 (80.9%)	43.77
Controls	Hypothyroid	0 (0%)	5.77
Controls	Non-hypothyroid	84 (100%)	78.23

Odds ratio

The conventional OR was undefined due to the zero cells in the control group. Following the Haldane-Anscombe correction (Tables 2, 3), the corrected OR was 14.88, indicating that hypothyroid individuals were approximately 15 times more likely to develop gallstone disease than euthyroid individuals.

**Table 2:** Contingency table: actual cell counts Total cases = 47; Total controls = 84

Group	Hypothyroid (n)	Non-hypothyroid (n)	Total
Cases	9	41	47
Controls	0	84	84

**Table 3:** Contingency table: after Haldane-Anscombe correction (+0.5 to all cells) Corrected OR = 14.88

Group	Hypothyroid	Non-hypothyroid
Cases	9.5	41.5
Controls	0.5	84.5

Gallstone composition

Mixed-type stones predominated in 93.6% of cases (n = 44), followed by cholesterol-type (4.3%, n = 2) and pigment-type (2.1%, n = 1). Of note, all nine hypothyroid cases had mixed-type stones (100% of hypothyroid cases), while the single pigment stone occurred in a euthyroid patient. This compositional clustering in hypothyroid patients is discussed in relation to the pathophysiology of bile cholesterol supersaturation in the Discussion section.

## Discussion

The principal finding of this study, a statistically significant association between hypothyroidism and gallstone disease (corrected OR 14.88, p = 0.00015), is consistent with a growing body of observational, experimental, and genetic-epidemiological evidence. To contextualize these findings for clinical practice and future research, we address five key domains below.

Age distribution

Classical epidemiological models predict rising gallstone prevalence after age 40, a pattern confirmed by the 2024 global meta-analysis of 32.6 million participants. The unexpected clustering of cases in the 30-39-year age group (48.9%, n = 23) therefore warrants explanation. Unnikrishnan et al. demonstrated that hypothyroidism in South India peaks in precisely the same 30-39 year bracket [6, 17], suggesting that accelerated lithogenesis driven by early-onset hypothyroidism may partially explain this pattern. Hypothyroidism-induced bile cholesterol supersaturation could accelerate stone formation in a younger demographic that would otherwise be at low classical risk [15]. 

Pathophysiology: the supersaturation-motility axis

Understanding why hypothyroidism promotes gallstone formation requires appreciation of the supersaturation-motility axis. At the hepatocyte level, thyroid hormone deficiency suppresses CYP7A1 expression, reducing conversion of cholesterol to bile acids and narrowing the bile acid pool. This drives biliary cholesterol supersaturation, which is the prerequisite for cholesterol crystal nucleation. At the gallbladder level, the absence of thyroxine’s pro-relaxation effect on the sphincter of Oddi increases biliary pressure and transit time [17-22]. Chen et al. confirmed that thyroid function causally influences cholelithiasis risk through lipid-mediated pathways [21].

Comparison with published Cureus and PubMed series

The 19.1% hypothyroidism prevalence in gallstone cases observed here compares closely with Mohammed Raza et al. [9], Laukkarinen et al. [18, 19], Kube et al. [23], Ravi et al. [24], and the 2024 Cureus cross-sectional study by Sinha and Prakash (30%) [25]. Higher estimates of 38.6%-40% have been reported in populations with elevated background thyroid dysfunction prevalence. In Indian surgical series, recent studies have reported hypothyroidism prevalence of 13.6-30% in gallstone patients, consistently exceeding matched controls.

Causal evidence and interpretation of the OR

The corrected OR of 14.88 exceeds values reported in some prior studies. Laukkarinen et al. reported an adjusted OR of 7.6 after controlling for age, sex, and BMI [12], and Volzke et al. found a 2.4-fold risk with TSH > 4.5 mIU/L in a large population-based cohort [13]. The higher estimate in this study likely reflects the smaller sample size and the mathematical properties of the Haldane-Anscombe correction, which inflates point estimates when zero cells are present. The direction and clinical significance of the association nevertheless remain consistent with the literature.

Crucially, two MR studies now provide causal evidence beyond observational association. Chen et al. established that thyroid function causally influences cholelithiasis through lipid-mediated pathways and confirmed a unidirectional causal effect of hypothyroidism on cholelithiasis [21].

Stone composition as a metabolic biomarker

The predominance of mixed stones (93.6%, n = 44) in this cohort is consistent with Indian surgical literature, where 76%-82% mixed stone prevalence reflects regional dietary habits and cholesterol metabolism [14, 22, 23]. The pathophysiological mechanism is straightforward: hypercholesterolemia in hypothyroidism drives bile cholesterol supersaturation, promoting nucleation and growth of cholesterol-rich stones. Inkinen et al. and Ravi et al. confirmed that in hypothyroidism, reduced bile flow and bile acid excretion produce cholesterol-dominant gallstones [15, 24]. The observation that all nine hypothyroid cases in this study had mixed-type stones, while the single pigment stone occurred in a euthyroid patient, provides direct compositional evidence linking thyroid status to stone chemistry. These clinical observations are now corroborated by Mendelian randomization evidence establishing a causal relationship from hypothyroidism to cholelithiasis [21]. And this corroborates the 2022 Cureus review finding that hypothyroid patients more frequently present with cholesterol-type or mixed stones [26]. Importantly, the 2024 Cureus study by Kulkarni et al. from Central India (n = 52) found hypothyroidism in 17.3% of gallstone patients and zero controls-a near-identical pattern to the present study, cross-validating its findings [27].

Clinical implications: revising the surgical workup

The mechanistic, observational, and causal evidence collectively supports incorporating routine TSH measurement into the standard surgical workup for gallstone disease. Hypothyroidism is an eminently treatable condition; levothyroxine replacement restores bile flow, gallbladder motility, and bile acid synthesis. This provides compelling justification for revising this guidance, especially in South Indian populations where background hypothyroidism prevalence is high [6].

Limitations

This study has several limitations that should be considered when interpreting findings. First, the case group fell below the pre-calculated sample size (47 achieved vs. 84 planned) due to COVID-19 restrictions, reducing statistical power and potentially inflating the corrected OR. Second, overt and subclinical hypothyroidism were not differentiated; failure to capture subclinical cases likely underestimates the true prevalence of thyroid dysfunction in this cohort. Third, important confounders, including body mass index, dyslipidemia, dietary habits, parity, and metabolic syndrome, were not systematically recorded, and residual confounding cannot be excluded. Fourth, gallstone composition was assessed by qualitative biochemical analysis rather than more precise techniques such as gas-liquid chromatography or infrared spectrophotometry. Fifth, the purposive non-random sampling strategy may introduce selection bias. Despite these constraints, the statistical significance of the primary finding remains robust, and the study contributes original prospective case-control data from South India to an area where such evidence is limited.

## Conclusions

This prospective case-control study demonstrates a strong, statistically significant association between hypothyroidism and gallstone disease in a South Indian tertiary-care cohort. Hypothyroid patients faced an approximately 15-fold elevated risk of cholelithiasis relative to euthyroid controls. The predominance of mixed-type stones and their exclusive occurrence in hypothyroid cases is mechanistically consistent with bile cholesterol supersaturation driven by thyroid hormone deficiency. These clinical observations are now corroborated by Mendelian randomization evidence establishing a causal relationship from hypothyroidism to cholelithiasis.

Routine TSH screening is recommended for all patients presenting with gallstone disease, particularly in populations with high background hypothyroidism prevalence. Early detection and treatment of thyroid dysfunction have the potential to mitigate lithogenesis and improve post-surgical outcomes. Future prospective multicenter studies with larger sample sizes, differentiation of subclinical and overt hypothyroidism, quantitative stone analysis, and systematic metabolic profiling are warranted to confirm these findings and evaluate whether levothyroxine therapy reduces gallstone recurrence after cholecystectomy.
